# Blame the Machine? Insights From an Experiment on Algorithm Aversion and Blame Avoidance in Computer-Aided Human Resource Management

**DOI:** 10.3389/fpsyg.2022.779028

**Published:** 2022-05-25

**Authors:** Christian Maasland, Kristina S. Weißmüller

**Affiliations:** ^1^Independent Researcher, Hamburg, Germany; ^2^KPM Center for Public Management, University of Bern, Bern, Switzerland

**Keywords:** algorithm aversion, blame avoidance, human resource management, algorithm-based decision support systems, behavioral experimental research

## Abstract

Algorithms have become increasingly relevant in supporting human resource (HR) management, but their application may entail psychological biases and unintended side effects on employee behavior. This study examines the effect of the type of HR decision (i.e., promoting or dismissing staff) on the likelihood of delegating these HR decisions to an algorithm-based decision support system. Based on prior research on algorithm aversion and blame avoidance, we conducted a quantitative online experiment using a 2×2 randomly controlled design with a sample of *N* = 288 highly educated young professionals and graduate students in Germany. This study partly replicates and substantially extends the methods and theoretical insights from a 2015 study by Dietvorst and colleagues. While we find that respondents exhibit a tendency of delegating presumably unpleasant HR tasks (i.e., dismissals) to the algorithm—rather than delegating promotions—this effect is highly conditional upon the opportunity to pretest the algorithm, as well as individuals’ level of trust in machine-based and human forecast. Respondents’ aversion to algorithms dominates blame avoidance by delegation. This study is the first to provide empirical evidence that the type of HR decision affects algorithm aversion only to a limited extent. Instead, it reveals the counterintuitive effect of algorithm pretesting and the relevance of confidence in forecast models in the context of algorithm-aided HRM, providing theoretical and practical insights.

## Introduction

With the rise of people analytics, human resource (HR) management today relies heavily on algorithm-based decision support systems to assist HR managers in the assessment of the value of individual employees as part of their organizations’ human capital asset (Reindl, 2016; Leicht-Deobald et al., 2019). People analytics is the creation of unique workforce insights by integrating originally disparate data sources from both inside and outside the organization to create HR insights with strategic value that lead to a competitive advantage.

While metric-driven analytical approaches to HR management date back to the beginning of the 20^th^ century (Huselid, 1995; Leicht-Deobald et al., 2019), quantifying individuals’ human capital as a specific asset to the firm has become much more sophisticated because of recent technological advances in algorithm-based machine learning and big data (Reindl, 2016). In a 2017 survey of 10,400 firms across 140 countries, Deloitte identified people analytics as one of the main global trends in human capital, with 71% of companies surveyed rating algorithm-based people analytics as a high priority in their organizations (Fineman et al., 2017). Today, a diverse landscape of software provides algorithm-based people analytic solutions for HR across all industries. For instance, Xerox Services have been using algorithm-based people analytics since as early as 2010 (Peck, 2013). Other key players in personnel information systems such as IBM, SAP, and Oracle use integrated application tools to accumulate HR data from existing databases (Angrave et al., 2016). Typical case studies of firms using algorithms strategically to enhance the efficiency of their talent management are, among many others, the tech giants Google (*People Analytics*; Shrivastava et al., 2018) and Microsoft (*MyAnalytics*; Giermindl et al., 2021), the bank ING (Peeters et al., 2020), the cybersecurity firm Juniper Networks (Boudreau and Rice, 2015), the retailer Wal-Mart (Haube, 2015), and online retailer Zalando using the software *Zonar* (Staab and Geschke, 2020).

Since the aforementioned workforce insights—generated by scoring employees based on computer-aided procedures—may inform HR practices not only descriptively but may also be used to predict future performance, applying people analytics raises substantial questions regarding legal and ethical matters (Leicht-Deobald et al., 2019), particularly regarding the quality of algorithmic predictions and their effect on HR managers’ choice architectures (Reindl, 2016). The idea of enhancing the quality of strategic HR management by using IT-based support systems that facilitate personnel decisions is deeply rooted in the paradigm of evidence-based management (Sharma and Sharma, 2017; Leicht-Deobald et al., 2019). In fact, the discourse on mechanical vs. clinical decision making shows that algorithms often outperform human forecast accuracy (Meehl, 1954; Sawyer, 1966; Kuncel et al., 2013). Yet, decision makers feel ambiguous about using algorithm-based support systems even though they know that doing so would optimize their choices and lead to objectively better outcomes (Grove and Meehl, 1996; Sanders and Manrodt, 2003; Fildes and Goodwin, 2007; Highhouse, 2008). Due to its relevance and rising popularity with decision makers in HRM (see, e.g., Sharma and Sharma, 2017), behavioral research on the effects of the availability of algorithm-based decision support systems on HR-related decision is highly relevant and has become a rapidly growing field of research lately (Burton et al., 2020).

As one of the first experimental studies in the field of algorithm aversion, Dietvorst et al. (2015) found that decision makers were reluctant to let their choice be guided by an algorithm-aided forecast model to determine which MBA students should be granted a scholarship although the quality and accuracy of the model forecast was clearly superior to human forecast ability. This phenomenon of *algorithm aversion* is intriguing because it contradicts the classic assumptions of utility maximizing behavior. While Dietvorst et al. (2015, 2018) successfully replicated their initial experiment, other studies further explored the effects of algorithm familiarity and trust (Berger et al., 2021; Filiz et al., 2021), algorithms’ characteristics such as their ability to learn (Berger et al., 2021), and their perceived fairness (Newman et al., 2020) as determinants of algorithm aversion, the underlying psychological mechanisms of algorithm aversion are still not explored sufficiently enough yet. Particularly, the effect of different choice types and valences on algorithm aversion is not well understood yet (but see initial findings by Newman et al., 2020, and Renier et al., 2021). Both studies by Dietvorst et al. (2015, 2018) are framed in a positive scenario of making performance evaluations with presumably rather positive valence (e.g., selecting students for a scholarship), but behavioral research on risk preferences (Kahneman and Tversky, 1979; Tversky and Kahneman, 1991) and blame avoidance in contextual frames (Vis and van Kersbergen, 2007; Bartling and Fischbacher, 2012) suggest that boundedly rational decision makers would react very differently when faced with the task of making decisions generally presumed to be unpleasant such as taking away the scholarship or dealing punishment. Little is known about whether people may prefer to delegate unpleasant choices to an algorithm to avoid blame and negative sentiment by shifting their personal responsibility to the machine (Langer and Landers, 2021). This is particularly relevant for HR management since HR managers regularly face tough workforce-related decisions—e.g., whom to lay off and whom to grant a promotion—while often being personally involved with their workforce. It is an obvious assumption that HR managers may be more likely to delegate making unpleasant HR decisions—e.g., laying off employees—to an algorithm compared to making more pleasant decisions (e.g., promoting employees) because the latter may entail less emotional burden and may offer the hedonic utility of making pleasant HR choices (Hamman et al., 2010). Does the type of HR choice task affect algorithm aversion?

The current study reports quantitative evidence from an original between-subject experimental study on the conditionality of algorithm aversion in positive and negative valence settings. Specifically, we report evidence from a 2×2 randomized controlled online vignette experiment conducted with a sample of *N* = 288 highly educated German residents. Set in the context of strategic HR management, we replicate prior experimental research by Dietvorst et al. (2015, 2018) and enhance their original experimental design by adding a contextual positive vis-à-vis negative affective vignette-based treatment (*personnel promotion* vs. *personnel dismissal*) to test whether decision makers are more likely to use algorithm-based decision support systems for making presumably unpleasant decisions—i.e., laying off staff—compared to presumably more pleasant decisions—i.e., promoting staff. We also control for the perceived algorithmic forecast precision, the role of pretesting it, and individuals’ confidence in human (CIH) and machine (CIM) forecast.

Following explicit calls by Dietvorst et al. (2015, 2018), Prahl and van Swol (2017), Tambe et al. (2019), and Newman et al. (2020), our research design comes with a few crucial methodological advantages. Its experimental setup with randomized controlled trials allows us to identify the latent causal mechanisms that relate algorithm aversion to blame avoidance based on the valence frame of a choice situation (i.e., promoting or dismissing staff). Our findings are directly relevant for HR management in practice because they add a new perspective to the scientific discourse on bounded rationality in the age of computer-aided choice architectures. This allows us to offer advice to HR managers in the form of caveats when using algorithms to enhance quality and precision in HR management.

In the next section, we review the literature on the motivational foundations of algorithm aversion and blame avoidance and derive two hypotheses on individuals’ likelihood to delegate critical HR decisions to algorithms in relation to individuals’ CIH and machine judgment. Then, we present the experimental design and procedure and report the results of the hypothesis testing with experimental data from *N* = 288 highly educated German respondents. We conclude with a discussion of the implications of our findings for theory and practice and suggest avenues for future research.

## Theory

### Origins of Algorithm Aversion

Algorithms are generally defined as a set of mathematical instructions that—without explicit human intervention—help calculate a solution to a given problem (Lee, 2018). Algorithms are based on elaborate statistical techniques that result in sophisticated forecasting models. In HR management, algorithm-based decision support systems profit from recent technical developments in machine learning and artificial intelligence that allow for high-level automatization in decision making “to supplement and inform (and perhaps supersede) human judgment or intuition” (Dietvorst et al., 2015; Prahl and van Swol, 2017; Lee, 2018).

Yet, empirical research across the whole spectrum of management science shows that decision makers are reluctant to use algorithms to maximize forecast precision and that people tend to discount machine-based forecast—compared to human-made forecast—even if explicitly informed about its superiority (Fildes and Hastings, 1994; Mentzer and Kahn, 1995; Dzindolet et al., 2002; Sanders and Manrodt, 2003; Fildes and Goodwin, 2007). For instance, Önkal et al. (2009) conducted a framing experiment with 130 graduate business administration students who were asked to predict stock prices. The experiment revealed that study participants discounted the perceived accuracy of a prediction presented as algorithm-based advice much more steeply compared to the case in which the very same prediction was presented as human-made. Medical, psychological, and financial studies also show that people generally prefer human judgment over machine-aided models of prediction and find human forecasts more trustworthy (Lee, 2018; Filiz et al., 2021). Research on computer-aided decision processes by Dzindolet et al. (2002) and Renier et al. (2021) shows that decision makers tend to perceive observed error rates of algorithm-based models as disproportionately more negative compared to human estimate-based models presumably because machine-made errors are incongruent to the widely held idea of perfection in machine-made forecast (Stangor and McMillan, 1992; Madhavan and Wiegmann, 2007; Boyd and Crawford, 2012; Constantiou and Kallinikos, 2015; Berger et al., 2021) and because negative information cues are psychologically more salient than positive information cues (Rozin and Royzman, 2001).

Prior studies on algorithm aversion hypothesized that there are a number of reasons for this preference for human-made forecast even in spite of explicit superiority of choices made by an algorithm: for instance, the notion that using algorithm-based choice modeling may be perceived as a loss of process ownership (Önkal and Gönül, 2005; Petropoulos et al., 2016), an abstract sense of unfamiliarity and hence distrust with the machine (Prahl and van Swol, 2017), or the notion that algorithms were unable to integrate qualitative factors (Grove and Meehl, 1996; Vrieze and Grove, 2009; Newman et al., 2020). Others argue that decision makers perceive algorithms as unable to account for unique and individual circumstances (Highhouse, 2008; Longoni et al., 2019), unable to deliver satisfying results in domains of high uncertainty (Dietvorst and Bharti, 2020), or mention machines’ lack of intuition and fairness (Newman et al., 2020), a quality typically associated with human forecasting (Lee, 2018; Burton et al., 2020). However, Dietvorst et al. (2015, 2018) were the first to conduct a series of experimental studies to identify the underlying behavioral mechanisms of algorithm aversion, especially concerning the correlation between the perceived fallibility of the algorithm-based choice models and the likelihood of delegating decisions to this algorithm. They found that decision makers were significantly less likely to delegate to the machine after seeing it perform (and inevitably err) even though the algorithm still dramatically outperformed their human judgment (Dietvorst et al., 2015, 2018). This effect was independent of the incentive structure, and their findings were replicated in follow-up studies by Prahl and van Swol (2017) and Dietvorst and Bharti (2020).

Recent studies have analyzed factors that may help reduce algorithm aversion. For instance, granting users limited control over the algorithm’s forecast reduced algorithm aversion (Dietvorst et al., 2018), and people tended to pardon an algorithm’s error if it was small and the decision domain was relatively predictable (Dietvorst and Bharti, 2020). Lee (2018) analyzed the acceptance rate of algorithm-based choices for tasks that require mechanical as opposed to human skills, and Castelo et al. (2019) found that increasing task objectivity and the human semblance of an algorithm’s pattern of decision making lead to higher trust in the algorithm. Yet to date, the underlying behavioral mechanisms of algorithm aversion regarding distinct types of decisions are still unexplored.

### Responsibility Shifting and Blame Avoidance

Prior studies exploring how people cope with making challenging or nonsocially acceptable decisions showed that by delegating a decision, individuals indeed shift both the mental burden and the factual or perceived responsibility for making the decision. Bartling and Fischbacher (2012) and Oexl and Grossman (2013) examined this psychological shifting process and showed that individuals affected by the outcome of a decision will in fact not blame the person responsible for making the decision but the person executing and delivering it. They stress that blame avoidance by shifting responsibility is a major motive for delegating unpopular decisions (see also Hill, 2015). Another example is the study by Erat (2013) that revealed that people will deliberately delegate the act of lying to their subordinates to avoid the responsibility for lying because such behavior is associated with negative sentiment, arousing psychological burdens in the form of hedonic disutility and the risk of blame. This finding corresponds with prior studies showing that individuals seek to avoid situations in which they may harm others, because decision delegation reduces decision makers’ mental costs of feeling responsible (Steffel et al., 2016). These psychological and moral factors relating to accountability are important predictors of algorithm aversion (Giermindl et al., 2021). For instance, experimental research by Newman et al. (2020) shows that people perceive algorithm-made HR decisions as less fair, a finding that was stable irrespective of whether employees were selected for promotion or layoff.

Given that the discourse on algorithm aversion suggests that delegating to an algorithm reduces perceived process ownership (Önkal and Gönül, 2005; Petropoulos et al., 2016), and given that the discourse on blame avoidance suggests reduced ownership is a behavioral strategy to cope with mental burdens in challenging choice situations, we hypothesize that the likelihood of delegating a HR decision to an algorithm is task dependent in the sense that:

*Hypothesis 1 (H1)*: Decision makers are more likely to delegate the decision of dismissing employees to an algorithm compared with promoting employees.

Yet, prior research on algorithm aversion (Dietvorst et al., 2015, 2018; Dietvorst and Bharti, 2020) indicates that individuals will be reluctant to delegate decision making to an algorithm if they feel its forecasting error is too high, rendering it unreliable. Making a HR decisions that may potentially change an employee’s life is a challenging situation, particularly for diligent HR managers. In the era of people analytics, HR decision makers are faced with peculiar moral conflict (Tambe et al., 2019): weighing the potential benefits and costs of delegating to an algorithm-based decision support system reduces individual mental costs by reducing perceive accountability and subjective expected blame but knowingly using a flawed algorithm may pose a violation of decision makers’ moral concept of self as an ethical and virtuous (i.e., “*blameless*”) person—particularly since many people assume that algorithm-based HR decision are less fair (Newman et al., 2020). The preservation of one’s moral concept of self is a psychological motive with high priority that strongly affects choice behavior (Bem, 1972; Hsee, 1996). Pretesting any genuine algorithm will reveal the realistic limits of its predictive quality and, hence, decision makers’ capacity to justify using it because “imperfect” algorithms violate the widely held expectation of software infallibility (Boyd and Crawford, 2012; Dietvorst and Bharti, 2020). This is why, among others, Dietvorst and Bharti (2020) and Renier et al. (2021), suggest that pretesting an algorithm reduces the likelihood of using it, suggesting that:

*Hypothesis 2a (H2a)*: Pretesting an algorithm reduces the likelihood of delegating HR decisions to the algorithm.

However, the perceived costs associated with delegating to a pretested algorithm may yet be conditional upon the type and valence of the choice to be delegated (Langer and Landers, 2021). While Newman et al. (2020) found that HR decisions made by an algorithm were perceived as less fair in both promotion and layoff decisions, the effect size of perceived human–machine difference in decision quality was lower for negative valence (i.e., layoffs) vis-à-vis positive valence (i.e., promotion) choice tasks. This points toward a potential asymmetry of the pretesting-related negativity bias toward the algorithm. However, recent empirical studies show that the degree of decision makers’ reaction to pretesting an algorithm may be conditional upon the quality of this pretesting experience. The perceived relative precision of the machine vis-à-vis human forecast precision may influence decision makers’ response (Filiz et al., 2021), as well as the specific task characteristic, particularly if stakes are high with regards to both tangible and moral costs (e.g., in the form of mental burdens; Lee, 2018; Burton et al., 2020). While research into this nexus is yet inconclusive (Langer and Landers, 2021), Renier et al. (2021) reveal that algorithmic fallibility will trigger harsher and comparatively more negative psychological reactions compared to experiencing equivalent human error and that a high-stakes HR choice context may be particularly salient in determining the acceptability of using an “imperfect” algorithm. Taken together, these initial findings on topical asymmetries suggest an alternative hypothesis.

*Hypothesis 2b (H2b)*: Pretesting the algorithm reduces the relative likelihood of delegating the decision of dismissing employees to an algorithm compared with promoting employees.

## Materials and Methods

### Experimental Design and Sample

The current study investigates whether people are more likely to delegate HR decisions to a computer algorithm if their decision is related to laying off employees compared to promoting employees. To test our hypotheses, we conducted a quantitative study using an interactive and dynamic online experiment in a randomized controlled 2×2 vignette design following best-practice advice by Atzmüller and Steiner (2010) and Aguinis and Bradley (2014) to warrant high levels of internal and external validity and to minimize social desirability-related response bias (Fisher and Katz, 2000). Based on actual HR decision support systems, we designed two equivalent yet topically opposite vignette scenarios—one dealing with making decisions on *promoting employees* (P) and one dealing with *dismissing employees* (D)—both of which offered respondents the opportunity to delegate the decision to a computer algorithm specifically designed to support these tasks. The two experimental conditions were corroborated with two alternative treatments to replicate algorithm aversion experiment of Dietvorst et al. (2015). The first treatment encompasses *pretesting* the algorithm before making the delegation choice (*pretest*; pt). The second treatment offered *no* option for *pretesting* the algorithm (nt). Consequently, the experiment defines four treatment groups (P__nt_, D__nt_, P__pt_, and D__pt_) based on two independent stimuli (choice task vignette condition *P* or *D* and treatment *nt* or *pt*) and a single dependent variable *delegation choice*, i.e., the decision of delegating the HR decision to the algorithm.

The online experiment was conducted between March and May 2018 and took between 20 and 30 min to complete. A convenience sample was raised by distributing the link to the experiment *via* several universities’ e-mail lists addressed to young professionals and graduate students and through online career networks, reaching a total of 574 individuals of which *N* = 288 (50.2%) fully completed the experiment. Respondents were incentivized by the prospect of winning one of several gift vouchers (€25, €50, or €75) for a popular online retailer. To warrant rigor, only complete responses were included in the final dataset of this study. The final sample comprises 156 (54.2%) women, 115 (39.9%) men, and 17 (5.9%) individuals who did not disclose. Respondents are on average *M* = 28.03 (*SD* = 6.1) years old, predominantly German citizens (91.0%), and highly educated with 169 (58.7%) having completed a tertiary degree education.

### Experimental Procedure

**Figure 1:**
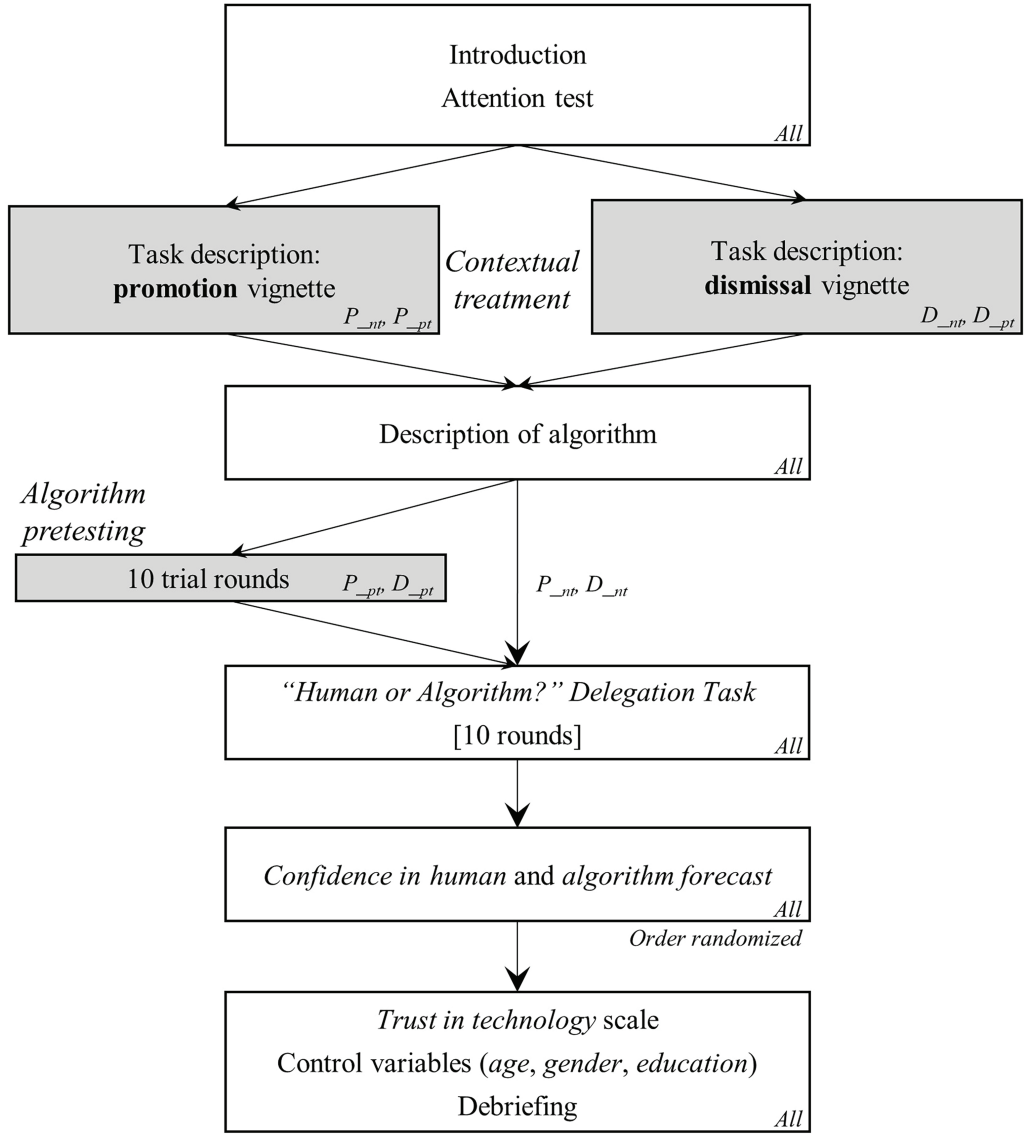
Experimental procedure.

Figure 1 provides an overview of the experimental procedure. Since the current study partially replicates the experiments conducted by Dietvorst et al. (2015, 2018), our vignette design and treatment wording were designed to resemble the former studies’ procedures as closely as possible. The original procedures were enhanced by adding the two different HR-related task stimuli—i.e., *promotion* or *dismissal*—to the vignette treatment, resulting in the 2×2 design.

First, respondents were introduced to the experimental setting placed in the context of employee performance evaluation. Participants were randomly sorted into one of the four treatment conditions and introduced to their role and respective tasks in the vignette scenario of this study. Respondents were asked to imagine being the *Chief Information Officer* (CIO) of a big company that employs hundreds of software engineers and to evaluate how successful current software engineers might be as software consultants in the future in comparison to other employees of the company’s workforce. In the promotion vignette, respondents were informed that their task was to predict the future performance of ten employees based on eight carefully selected criteria typical for software engineers. They were told that these employees would later be ranked against each other and that all employees who scored above a certain but unknown threshold would be promoted.^1^ Similarly, in the dismissal vignette, respondents presented with the identical criteria and information were informed that their predictions regarding employees’ future performance was to be used to determine that employees who scored below a certain unknown threshold level would be dismissed. These conditions provide a balanced equivalent treatment framed in two different choice scenarios.

Next, all participants were informed that they were free to use an algorithm-based decision support system, which would produce forecasts based on the very same data available to the respondents themselves. Participants were given further information to confirm that it was a sophisticated algorithm created by diligent expert analysts.

Then, study participants were, again, randomly sorted into the *pretest* (pt) and the *no-test* (nt) condition branch of the experiment. Respondents in the pretest condition were asked to make 10 practice rounds, in which they could see the algorithm perform before they would make the 10 employee predictions, which would also determine their likelihood of winning the incentive lottery. In each of the 10 trial rounds, respondents were informed about employees’ “real” performance score (range: 1–100) and the performance score predicted by the algorithm, respectively. Respondents sorted into the no-test condition directly moved on to their task, i.e., they had no chance to see the algorithm perform *a priori*.^2^ In this context, it is important to note that the algorithm’s absolute average prediction errors (AAE) across all trials were designed following procedure of Dietvorst et al. (2015) to ensure that human and algorithmic forecast accuracy were virtually identical across all conditions, vignettes, and rounds. This component is an important design aspect to warrant that there is no quality-related, functional reason to disregard algorithm support. The main dependent variable of this experiment is whether respondents choose to make the forecast themselves (*delegation choice* = 0) or delegate their task to the algorithm (*delegation choice* = 1). Respondents were randomly presented with 10 vignettes drawn from the set of 20 employee vignettes and asked to predict each employee’s future performance on a scale from 1 to 100. Prior to these 10 rounds, respondents were asked whether they would like to tie their predictions to those made by the algorithm. During these 10 rounds, participants saw no information about employees’ “true” performance scores. After completing the 10 rounds, respondents were asked to indicate their confidence in human forecast and their confidence in the algorithm forecast, respectively, on a five-point Likert-type scale ranging from 1 = “*no confidence*” to 5 = “*full confidence*.” We use this *post-hoc* measure for explorative analyses.

As a control variable, respondents’ *trust in technology* was measured after to the experimental tasks with seven-item Likert-type measure of McKnight et al. (2011) on trust in information technology. The original English scale items were translated into German with due diligence. Furthermore, respondents were asked to indicate their *age*, *gender*, *level of education*, and *country of residence*, in each case allowing for nonresponse, before being debriefed.

## Results

### Descriptive Analysis

The four vignette treatments were randomly distributed among the study participants. As not all respondents finished the experiment, the treatment distribution varies; the overall distributions of treatments are n_P_nt_ = 77 (26.7%), n_D_nt_ = 81 (28.1%), n_P_pt_ = 59 (20.5%), and n_D_pt_ = 71 (24.7%). Of the final sample, 136 (47.2%) received the positive *promotion* frame (P) and 152 (52.8%) of the final sample received the negative *dismissal* frame (D), 158 (54.9%) people received the no-test condition (nt) and 130 (45.1%) the pretest condition (pt).

Table 1 displays the correlation matrix of all variables. For the current sample, the bidimensional *trust in technology* measure of McKnight et al. (2011) resulted in a satisfying level of construct reliability [faith in technology (general): *α_fit_* = 0.61; trusting stance: *α_ts_* = 0.69]. Of all *N* = 288 respondents, *n* = 66 (23.0%) chose to use the algorithm. Logistic regression modeling with [*χ*^2^(8) = 55.29, *p* < 0.000; pseudo-*R*^2^ = 0.200] and without control variables [*χ*^2^(4) = 56.78, *p* < 0.000; pseudo-*R*^2^ = 0.195] reveals that neither respondents’ trust in technology, age, gender, nor education explained any substantial amount of variance in choice. However, individuals’ confidence in human and machine forecast do influence the dependent variable. We explore this finding in *post-hoc* analyses after hypothesis testing below.

**Table 1:** Correlation matrix.

	Variable	1		2		3		4		5		6	7			8	
1	Delegation choice [0, 1] = [human decision; algorithm’s decision]	–															
2	Algorithm pretested? [0, 1] = [no; yes]	−0.18_a_^**^		–													
3	HR choice task [0, 1] = [promotion; dismissal]	0.05_a_		0.03_a_		–											
	Confidence in…																
4	…Algorithm forecast	0.36_a_^***^		−0.15_a_^*^		−0.08_a_		–									
5	…Human forecast	−0.25_a_^***^		−0.02_a_		−0.04_a_		−0.12_c_^*^		–							
6	Trust in technology	0.06_b_		−0.08_b_		0.03_b_		0.17_c_^**^		0.08_c_		–					
7	Age (years)	−0.01_b_		−0.06_b_		0.06_b_		0.04_c_		−0.06_c_		0.03		–			
8	Female	−0.08_a_		0.08_a_		−0.05_a_		−0.05_a_		−0.02_a_		−0.10_b_		−0.08_b_		–	
9	Tertiary education	0.03_a_		0.01_a_		0.14_a_^*^		0.01_c_		0.01_c_		0.07_c_		0.06_c_		0.06_a_	

a = Cramer’s *φ*_c_; b = Point biserial correlation r_pb_; and c = pearson’s r.

* *p* < 0.05;

** *p* < 0.01;

*** *p* < 0.001.

Participants in the no-test condition assumed that the algorithm-based forecasting model was more accurate (*M* = 40.6 ± 26.4) compared with participants in the pretest condition (*M* = 32.82 ± 22.3); Welch’s *t*(271.65) = 2.64, *p* < 0.01, *d* = 0.32. Confidence in the algorithm is significantly associated with a higher likelihood of delegating the decision to the algorithm (*φ*_c_ = 0.36, *p* < 0.000), while confidence in the human forecast is negatively correlated with the likelihood of delegating to the algorithm (*φ*_c_ = –0.25, *p* < 0.000). This effect is asymmetric in the sense that higher confidence in the algorithm has a higher positive effect than higher confidence in human forecast has a negative one. Pretesting the algorithm (i.e., seeing it perform and, inevitably, err) has a negative effect on the likelihood of delegating to the algorithm (*φ* = −0.18, *p* < 0.01). This is an astonishing finding given that the experiment was designed so that prediction accuracy—i.e., the absolute average prediction error (AAE)—of the human and the algorithmic forecast were virtually identical, with the algorithm’s AAE (*M* = 20.02 ± 1.56) being on average even smaller than the human AAE (*M* = 20.71 ± 5.01) on the 1–100 performance score; *t*(352.4) = −2.19, *p* < 0.05 (see Appendix C for more detailed analyses).

### Hypotheses Testing

**Figure 2:**
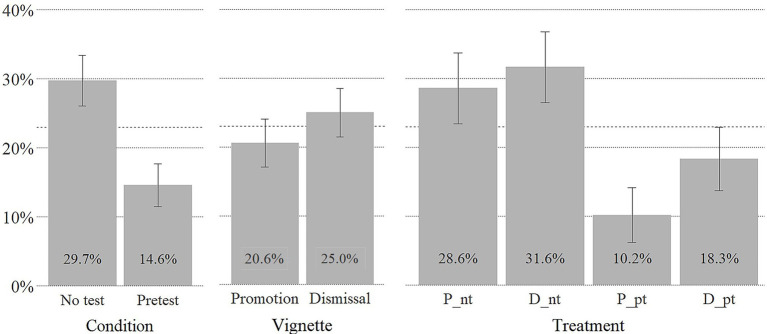
Share (S) of participants delegating human resource (HR) decision to algorithm. Error bars denote ±1 SE; dashed line indicates sample mean of 23%.

Figure 2 displays the share (*S*) of participants who delegated their decision to the algorithm split by condition, vignette, and treatment. We find that participants in the no-test condition and participants in the dismissal vignette scenario were more likely to choose to let the algorithm make the decision. Being able to pretest the quality of the algorithm (see Figure 2) [*ΔS*(*Algorithm pretesting*) = *S*(no-test) – *S*(pretest) = 15.1%] had a bigger effect on choice than the type of HR decision [Δ*S*(*decision type*) = *S*(Dismissal) – *S*(Promotion) = 4.4%]. Participants in the *promotion and pretest treatment* (*P__pt_*) were least likely to delegate to the algorithm [*S*(P_*_*pt_) = 10.2%] and participants in the *dismissal and no-test treatment* (*D_*_*nt_*) were most likely to delegate to the algorithm [*S*(D_*_*nt_) = 31.6%].

Treatment groups had unequal sizes and were nonnormally distributed but variances were distributed homogenously [Levene’s test *F*(3, 270) = 2.03, *p* = 0.11]. Table 2 presents the odds ratios of delegating to the algorithm by treatment condition. We find no task-related treatment effects purely in relation to dismissing vis-à-vis promoting employees [*χ*^2^_D_nt/P_nt_ (1) = 0.10, *p* = 0.75, *φ*_D_nt/P_nt_ = 0.03; *χ*^2^_D_pt/P_pt_ (1) = 1.71, *p* = 0.19, *φ*_D_pt/P_pt_ = 0.11]. Consequently, H1 finds no support.

**Table 2:** Odds ratios, *χ*^2^-, and *φ*-tests of choice by condition, type, and treatment.

	Condition		HR choice type		Treatment			
	No-test	Pretest	Promotion	Dismissal	P__nt_	D__nt_	P__pt_	D__pt_
*n*	158	130	136	152	77	81	59	71
*n_Model_*	47	19	28	38	22	25	6	13
*n_Human_*	111	111	108	114	55	56	53	58
Odds_Model_	0.42	0.17	0.26	0.33	0.40	0.45	0.11	0.22
Odds_Human_	2.36	5.84	3.86	3.00	2.50	2.24	8.83	4.46
∆Odds_Model_ [95% CI]	∆Odds_pt/nt_ = 0.41 [0.21; 0.76]		∆Odds_D/P_ = 1.28 [0.71; 2.34]		∆Odds_P_pt/P_nt_ = 0.29 [0.09; 0.80]			
					∆Odds_D_pt/D_nt_ = 0.5 [0.21; 1.14]			
					∆Odds_D_nt/P_nt_ = 1.12 [0.53; 2.34]			
					∆Odds_D_pt/P_pt_ = 1.97 [0.64; 6.79]			
*χ*^2^-tests	*χ*^2^_pt/pt_(1) = 9.24, p < 0.01		χ^2^_D/P_(1) = 0.79, p = 0.37		*χ*^2^_P_nt/P_pt_ (1) = 6.92, *p* < 0.01			
					*χ*^2^_D_nt/D_pt_ (1) = 3.18, *p* = 0.08			
					*χ*^2^_D_nt/P_nt_ (1) = 0.10, *p* = 0.75			
					*χ*^2^_D_pt/P_pt_ (1) = 1.71, *p* = 0.19			
*φ*	*φ*_nt/pt_ = −0.18^*^		*φ*_D/P_ = 0.05		*φ*_P_nt/P_pt_ = −0.23^*^			
					*φ*_D_nt/D_pt_ = −0.14			
					*φ*_D_nt/P_nt_ = 0.03			
					*φ*_D_pt/P_pt_ = 0.11			

* *p* < 0.05.

However, pretesting the algorithm has a significant effect on the likelihood of delegating the HR decision to the algorithm, irrespective of choice type: Pretesting the algorithm reduces the likelihood of delegating an HR decision to the algorithm [*χ*^2^_nt/pt_(1) = 9.24, *p* < 0.01, *φ*_nt/pt_ = −0.18*]. This effect is stable across both choice task treatments but stronger in the setting of selecting employees for promotion [*χ*^2^_P_nt/P_pt_(1) = 6.92, *p* < 0.01, *φ*_P_nt/P_pt_ = −0.23*] than for dismissal [*χ*^2^_D_nt/D_pt_(1) = 3.18, *p* = 0.08, *φ*_D_nt/D_pt_ = −0.14]. However, only the effect of the promotion-related setting is statistically significant and reliable (*φ*_P_nt/P_pt_ = −0.23*). We investigate the interaction between the valence type of the HR decision task and pretesting further by conduction logistic regression analyses (see Model III in Table 3) and find no statistically significant interaction between the promotion and dismissal choice context (odds ratio: 0.609, *p* = 0.502). This means that pretesting the algorithm reduces decision makers’ likelihood of delegating to the algorithm, irrespective of the choice contest, supporting the baseline hypothesis H2a but not H2b.

**Table 3:** Logistic regression results on choice to delegate HR decision to algorithm.

	Model I				Model II				Model III			
	Odds ratio	SE	[95% CI]		Odds ratio	SE	[95% CI]		Odds ratio	SE	[95% CI]	
*Treatment effects*												
Pretesting the algorithm	0.448^*^	0.159	0.223	0.900					0.546	0.250	0.223	1.339
HR choice type: dismissal	0.589	0.211	0.291	1.190					0.703	0.311	0.295	1.675
*Combined treatment effects*												
Promotion and pretest (P_pt)					*– reference category –*							
Dismissal and pretest (D_pt)					2.336	1.410	0.716	7.624				
Dismissal and no pretest (D_nt)					3.007^†^	1.742	0.966	9.357				
Promotion and no pretest (P_nt)					4.278^*^	2.472	1.378	13.280				
*Interaction effects*												
Pretesting × dismissal									0.609	0.450	0.143	2.588
*Control variables*												
Confidence in algorithm forecast	2.483^***^	0.490	1.687	3.655	2.478^***^	0.490	1.683	3.650	2.478^***^	0.490	1.683	3.645
Confidence in human forecast	0.447^***^	0.099	0.290	0.690	0.451^***^	0.100	0.292	0.697	0.451^***^	0.100	0.292	0.697
Trust in technology	0.969	0.326	0.501	1.873	0.993	0.336	0.511	1.927	0.993	0.336	0.511	1.927
Age	0.993	0.029	0.938	1.052	0.994	0.029	0.939	1.052	0.994	0.029	0.939	1.052
Female	0.916	0.326	0.456	1.841	0.927	0.331	0.460	1.866	0.927	0.331	0.460	1.867
Higher education	1.108	0.423	0.524	2.342	1.105	0.422	0.523	2.335	1.105	0.422	0.523	2.335
*Constant*	0.511	0.677	0.038	6.862	0.099^†^	0.135	0.007	1.414	0.425	0.576	0.030	6.053
*N*	267				267				267			
LR *χ*^2^(df)	55.29^***^				55.75^***^				55.75^***^			
df	8				9				9			
Pseudo-*R*^2^	0.200				0.201				0.201			
Log likelihood	−110.81				−110.58				−110.58			

*Post-hoc* analyses.

† *p* < 0.10.

* *p* < 0.05;

*** *p* < 0.001.

### Explorative Analyses

Since the effect of algorithm aversion is so prevalent in our data, we conduct *post-hoc* analysis to further investigate the role of CIH vis-à-vis machine-based (CIM) forecasting on individuals’ likelihood of delegating HR decisions to an algorithm. Correlation analysis (Table 1) revealed that both forms of confidence affect the likelihood of delegation: Higher confidence in machine-based forecasting is correlated with a higher likelihood of delegating to the algorithm (*φ*_c_ = 0.36, *p* < 0.000), and pretesting it reduces the confidence in machine-based forecast (*φ*_c_ = −0.18, *p* < 0.000). Similarly, higher confidence in human forecasting is significantly correlated with a lower likelihood of delegating an HR decision to an algorithm (*φ*_c_ = −0.25, *p* < 0.000).

**Figure 3:**
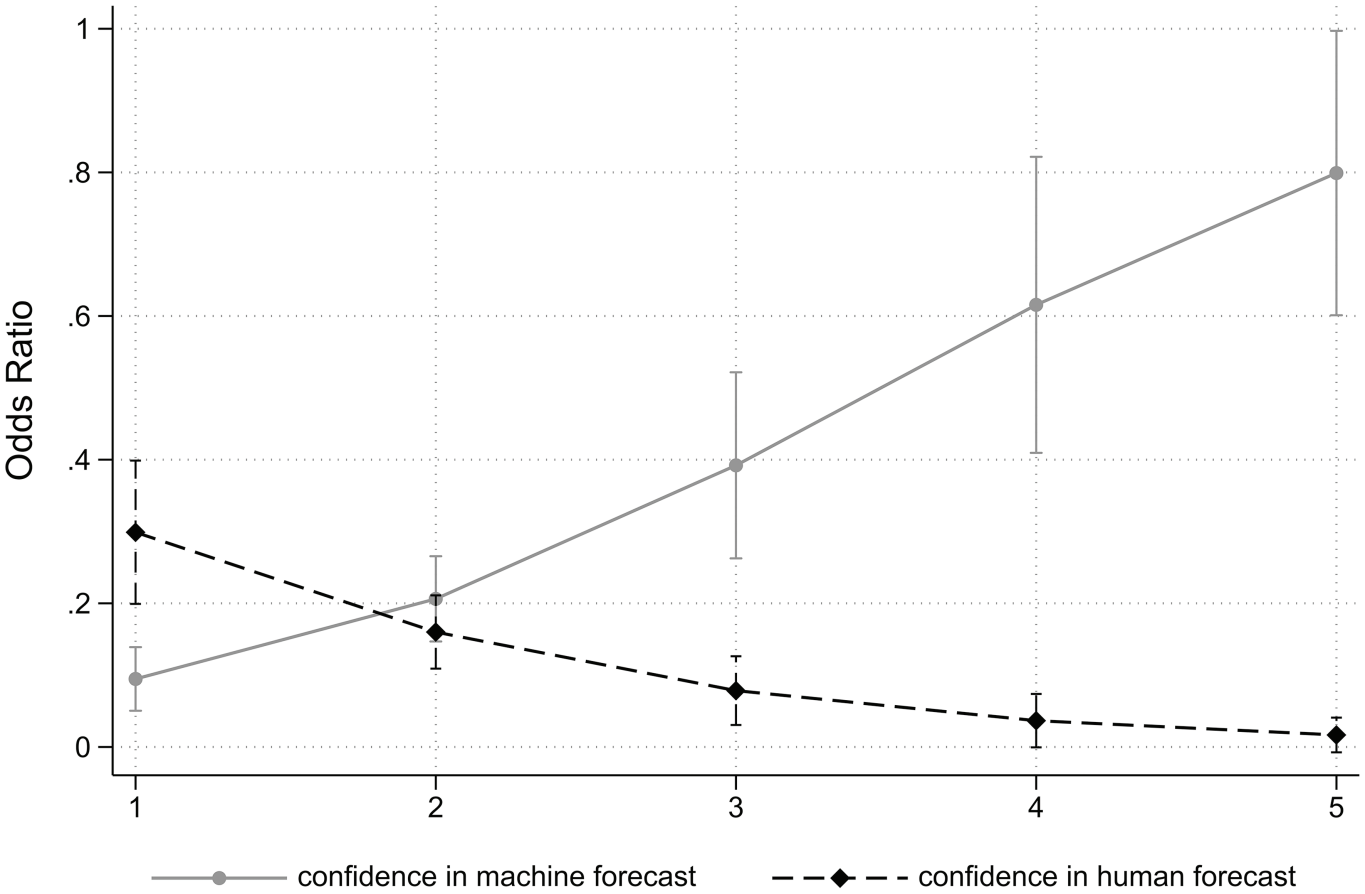
Marginal effects plot of confidence in human (CIH) and machine (CIM) forecast on choice to delegate HR decision to algorithm.

Logistic regression was used to further analyze the relationship between pretesting the algorithm, choice frame, CIM, and CIH on the probability of delegating the HR decision to the algorithm (see Table 3). As displayed in Model I of Table 3, we find that, holding the other variables constant, the odds of delegating to the algorithm decreased by 55.2% (95% CI [0.223, 0.900]; *p* = 0.024) for study participants who pretested the algorithm. In the dismissal choice frame, the odds of delegating to the algorithm decreased by 41.1% (95% CI [0.291, 1.190]) but this effect is not statistically significant (*p* = 0.140), supporting the findings presented in the previous section. Furthermore, confidence in the machine forecast (CIM) dramatically increases the odds of delegating to the algorithm (odds ratio: 2.483, *p* < 0.000). For each marginal increase on the five-point CIM-scale, individuals were 148% (95% CI [1.687, 3.655]) more likely to delegate. While confidence in human forecast (CIH) also significantly influences the choice to delegate, its effect is smaller. For each marginal increase in CIH, the likelihood of delegating to the algorithm decreased by 55.3% (95% CI [0.290, 0.690]), these divergent marginal effects are illustrated in Figure 3. In Model II, we investigate the relation between the combined treatment effects (HR choice type and pretest condition) but the effects of the confidence variables CIH and CIM remain equally strong. Further analysis revealed no other significant interaction effects.^3^

**Figure 4:**
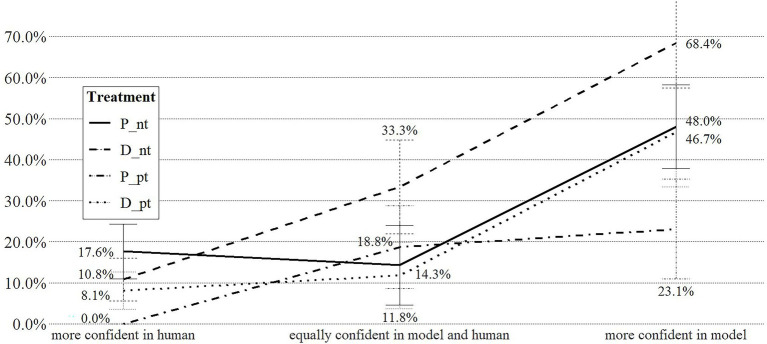
Share (S) of participants delegating HR decision to algorithm, by treatment and confidence levels.

Since individuals may hold divergent levels of confidence in human and machine forecast, we illustrate their effect on the choice to delegate the HR decision to the algorithm further in Figure 4, which displays the share of respondents delegating their HR decision to the algorithm by treatment and clustered by individual confidence levels to account for individual differences in confidence configurations. Individuals who are more confident in human vis-à-vis algorithm-based decision making are less likely to delegate their decisions (0.0–17.6% of respondents). While variation within this group exists in relation to the type of choice task (dismissing vis-à-vis promoting employees) and pretesting the algorithm (test vis-à-vis no-test), the differences are not statistically significant. Among respondents equally confident in model and human forecast, only 14.3% delegate dismissal decisions to the algorithm if they had the chance to pretest it, but 33.3% do so if they did not pretest the algorithm. We find no equivalent discrepancy for the promotion scenario (P_pt = 18.8%; P_nt = 14.3%). Individuals more confident in machine-based forecasts exhibit the strongest effects. Of this group of respondents, 68.4% will delegate dismissal decisions to an algorithm, but only 46.7% do so if they had a chance to pretest the algorithm. Similarly, 48.0% individuals with relatively higher confidence in machine-based decision making will delegate promotion decisions to the algorithm, but only 23.1% do so after testing the algorithm.

## Discussion

The experimental findings reveal that changing the valence type of HR decision from a presumably unpleasant decision (i.e., dismissing staff) to a presumably preferable one (i.e., promoting staff) does not necessarily affect the likelihood of delegating this choice to a supportive algorithm. The absence of a substantial direct effect in a strictly controlled experimental study with a relevant sample of young professionals and graduate students has important implications for human resource management scholarship and practice: It shows that the affective reference frame of an HR decision does not function unconditionally as a reliable predictor for whether decision makers will use algorithmic decision support systems to avoid blame and shift the mental burden of responsibility. While there is some indicative support for a choice type-related effect, this effect is much weaker than anticipated and conditional upon the perceived quality of the algorithm forecast as well as decision makers’ confidence in machine vis-à-vis human forecast quality. People will not automatically use the algorithm to shift blame.

This is the first experimental study to investigate algorithm aversion and blame avoidance in HR using a German sample. We find the same direction of effects as Dietvorst et al. (2015, 2018) and have hence replicated and extended their results. The study of Dietvorst et al. (2015) was conducted with MBA students from a United States university, while our study relies on experimental data raised with a sample of both young professionals and graduate students from Germany. We contribute to the generalizability of the findings regarding algorithm aversion internationally and in practice, complementing recent scholarship by, among others, Lee (2018), Newman et al. (2020), Filiz et al. (2021), and Renier et al. (2021). Although our findings on algorithm aversion are statistically significant and robust, the effect sizes we observe are substantially smaller—i.e., only one third as large as in Dietvorst et al. (2015). One explanation for the differences in effect sizes is that country cultures exhibit specific differences in trust in artificial intelligence (Gillespie et al., 2021).

The experimental evidence of our study relies on an innovative, balanced, and randomly controlled experiment to warrant high internal validity and to eradicate the influence of socio-demographic factors, which might differentiate employees in their interaction with technology and prime their decision delegation likelihood (McKnight et al., 2011). Neither respondents’ trust in technology, age, gender, nor level of education, explained any substantial amount of variance in delegating choice. This is in line with study of Dietvorst et al. (2015), which did not find significant effects regarding these control variables either. This similarity underlines that our results are substantial regarding the observation that blame avoiding behavior is conditional while algorithm aversion is a fundamental psychological mechanism.

Compared with participants in the pretest condition, participants in the no-test condition assumed that the algorithm-based forecasting model was more accurate. This means that testing—and thereby experiencing the fallibility of an algorithm—increases algorithm aversion rather than decreasing it. This finding is in line with prior empirical findings of Dietvorst et al. (2015, 2018), Burton et al. (2020), and Prahl and van Swol (2021), and it highlights practitioners’ peculiar challenges in encouraging the use of algorithm-based decision support systems in reluctant staff.

Supporting prior research on blame avoidance in other fields of decision research (Vis and van Kersbergen, 2007; Bartling and Fischbacher, 2012), we specifically contribute to HRM scholarship by revealing that study participants tasked with dismissing staff tend to delegate to the algorithm but only under certain conditions related to their confidence in human and machine forecast, echoing prior findings on the essential role of confidence in human and machine forecast by Filiz et al. (2021). In contrast, pretesting the algorithm reduces the likelihood of delegating an HR decision to it. This effect is stable across both types of HR decisions assessed, but it is stronger in the setting of selecting employees for promotion than for dismissal, all else being equal. However, only the effect of the promotion-related setting is statistically reliable. This implies that decision makers tend to be less likely to delegate presumably more pleasant promotion decisions to an algorithm, but this relationship is only statistically reliable if pretesting is possible. One explanation for this pattern is that choice delegation may indeed come with a loss of psychological ownership, which is affectively undesirable for (presumably) more pleasant tasks that involve hedonic utility for the decision maker (Önkal et al., 2009; Cassotti et al., 2012; Stark et al., 2017). It is, therefore, individually rational not to delegate if individuals perceive their decision to result in affectively pleasant outcomes in social contexts (Forgas, 2006). Another explanation relates to the flawed expectation of ultimate perfection in algorithms’ precision and performance when used in algorithm-based decision support systems in HR (Boyd and Crawford, 2012; Ziewitz, 2016). Individuals mostly expect algorithms to be infallible (Dietvorst et al., 2015; Dietvorst and Bharti, 2020; Berger et al., 2021). When confronted with algorithms’ realistic error margins, this expectation disconfirmation may trigger the psychological effect of dissatisfaction-based negativity bias (Oliver, 1980; Oliver and DeSarbo, 1988). This means that experiencing the potentially unexpected fallibility of an algorithm triggers an asymmetrically larger negative response than experiencing the strengths of the algorithm triggers a positive response, even though the algorithm still outperforms human forecast precision. Since recent research by Renier et al. (2021) support this presumption of algorithm-error related negativity bias, we assume that the widely held expectation of algorithm-based HR decision support systems as perfect (Constantiou and Kallinikos, 2015; Leicht-Deobald et al., 2019) may have caused respondents to experience the realistic imperfection of the algorithm in our experiment as a negative surprise, which may have increased reluctance to use it (Madhavan and Wiegmann, 2007; Prahl and van Swol, 2017; Lee, 2018).

We find that decision makers’ level of confidence in human vis-à-vis machine-based forecast is a strong predictor of their likelihood of delegation, which is in line with recent empirical findings by Berger et al. (2021). Higher confidence in machine-based forecasting is correlated with a higher likelihood of using the decision support system, which poses a paradoxical practical challenge because pretesting an “imperfect” (i.e., realistic) algorithm *reduces* the confidence in machine-based forecasting. This corresponds to prior findings by Lee (2018). Likewise, higher confidence in human forecasting is significantly correlated with a lower likelihood of delegating HR decisions to an algorithm. Individuals who are more confident in human vis-à-vis algorithm-based forecast precision have a very low likelihood of delegating the decision. Confidence in the algorithm is significantly associated with a higher likelihood of delegating the decision to the algorithm, whereas confidence in the human forecast is negatively correlated with the likelihood of delegating to the algorithm. This effect is asymmetric in the sense that higher confidence in the algorithm has a higher positive effect than higher confidence in human forecast has a negative one. This is in line with prior research on human–human vis-à-vis human–machine trust (Madhavan and Wiegmann, 2007; Prahl and van Swol, 2017, 2021; Filiz et al., 2021). Practitioners need to be aware that familiarity with an algorithm—i.e., the chance to pretest it—may lead to asymmetries in the likelihood of using the algorithm despite the algorithm’s usefulness and high forecast precision. Algorithm aversion is, under some conditions, choice task dependent (see also Castelo et al., 2019), but the nature of the choice task is not the decisive factor. While presumably more pleasant HR decisions such as promoting employees may reduce the likelihood of using algorithmic support it is important to note that the combination of pretesting, low confidence in machine forecast, and task type may lead to biased choices and unintended outcomes. Practitioners are encouraged to help their staff build organic trust in their decision support systems but also foster awareness of both the advantages but also risks in using algorithm-based help in HRM (Leicht-Deobald et al., 2019).

### Limitations and Future Research

As all empirical research, the generalizability of the findings of the current experiment is limited to some extent. First, while this study relies on data of a highly educated sample of individuals at the start of their careers, it is a convenience sample and not representative for the general population. Yet, in view of the aim of the study, the sample is relevant as it resembles the typical socio-demographic profile of university graduates that are highly in demand for diverse types of managerial training positions in Germany. Furthermore, we are confident in the generalizability of our results (within certain boundaries) since our study replicates and extends the results by Dietvorst et al. (2015).

We identify two specific avenues for future research. First, we believe that more experimental research—especially using realistic treatment conditions with an elevated level of respondent immersion—is needed to further explore the effect of perceived algorithm forecast accuracy as a necessary condition for blame avoidance. Our findings suggest that the likelihood of using an algorithm to support their HR decisions is contingent upon the type of decision but also confidence in, expectations toward, and prior interaction experience with algorithmic forecasting models. Future studies are encouraged to replicate and enhance our experimental design by systematically manipulating the quality of the algorithm to investigate whether the effect is linear or dynamic. Particularly, we encourage future research to replicate our research design with a specific focus on HR professionals’ and managers’ perspective to explore the effect of professional experience and confidence in human vis-à-vis machine-based forecasts in more detail.

Second, more research is needed to explore the phenomenon of the saturation effect of algorithm aversion. Our data reveal the same stable saturation level as Dietvorst et al. (2015), while using a sample from another country, i.e., Germany. Although this strongly indicates that there is a base-line psychological mechanism at work, more quantitative replication studies with samples from other countries, cultures, and HR tasks are needed to assess its generalizability.

## Data Availability Statement

The raw data supporting the conclusions of this article will be made available by the authors, without undue reservation.

## Ethics Statement

Ethical review and approval was not required for the study on human participants in accordance with the local legislation and institutional requirements. Written informed consent for participation was not required for this study in accordance with the national legislation and the institutional requirements.

## Author Contributions

All authors listed have made a substantial, direct, and intellectual contribution to the work and approved it for publication.

## Funding

Open access funding was provided by the University of Bern.

## Conflict of Interest

The authors declare that the research was conducted in the absence of any commercial or financial relationships that could be construed as a potential conflict of interest.

## Publisher’s Note

All claims expressed in this article are solely those of the authors and do not necessarily represent those of their affiliated organizations, or those of the publisher, the editors and the reviewers. Any product that may be evaluated in this article, or claim that may be made by its manufacturer, is not guaranteed or endorsed by the publisher.

## Footnotes

^1^These criteria were carefully designed to optimize the external validity of the experiment by maximizing respondents’ immersion into a highly realistic choice scenario. A more extensive description of the experiment and stimuli is presented in Appendix A and B.

^2^To inhibit halo and primacy effects (Shteingart et al., 2013), the employee-based stimuli were presented in random order between subjects and were drawn from a carefully designed sample of 20 software engineers to introduce sufficient amounts of variance (see also Appendix C).

^3^Additional results of interaction effects analyses are available from the authors upon request.
