# Erratum: Blame the machine? Insights from an experiment on algorithm aversion and blame avoidance in computer-aided human resource management

**DOI:** 10.3389/fpsyg.2023.1186486

**Published:** 2023-03-24

**Authors:** Frontiers Production Office

**Affiliations:** Frontiers Media SA, Lausanne, Switzerland

**Keywords:** algorithm aversion, blame avoidance, human resource management, algorithm-based decision support systems, behavioral experimental research

An omission to the funding section of the original article was made in error. The following sentence has been added: “Open access funding was provided by the University of Bern.”

The original article has been updated.

