# Supplementary material for: Blame the Machine? Insights From an Experiment on Algorithm Aversion and Blame Avoidance in Computer-Aided Human Resource Management
Source: Front Psychol. 2022 May 25;13:779028. doi: 10.3389/fpsyg.2022.779028 (PMC9177159; doi:10.3389/fpsyg.2022.779028)
Supplement: Supplementary file 1 [file Data_Sheet_1.pdf]

Online Appendix – Supplementary Material

to

Blame the Machine?

Insights from an Experiment on Algorithm Aversion and Blame Avoidance in Computer-Aided Human Resource Management

by

Christian Maasland & Kristina S. Weißmüller

Original Research published in:

Frontiers in Psychology, 2022, Vol. 13: 779028. doi: 10.3389/fpsyg.2022.779028;

https://www.frontiersin.org/articles/10.3389/fpsyg.2022.779028/full

Online Appendix – Supplementary Material

Appendix A – Experimental Codebook and Questionnaire

Appendix B – Treatment and Ranking Mechanism

Appendix C – Model and Human Absolute Average Prediction Error *M* (*SD*)

Appendix A – Experimental Codebook and Questionnaire

Notes: Extensive codebook and original German version upon request. *P* = Promotion; *D* = Dismissal; *pt* = pretest; *nt* = no-test

No.	Text
1	**[Introduction]**<br><br>"Dear Participant,<br><br>Thank you very much for taking part in this study. You are about to join a scientific online experiment on 'Employee Evaluation'. Please read and answer the following questions calmly and carefully and reply as honestly as you can.<br><br>The study should take you approximately 20-25 minutes to complete. Please be assured that your responses will be made anonymous and used for this research project only. By participating, you actively support an important non-profit research project at the University of XY. Thank you very much! (Dietvorst et al., 2015)<br><br>All participants have the chance to win one of three gift vouchers (1 x 75€, 1 x 50€, 1 x 25€). If you would like to take part in the awarding, please provide your email address at the very end of this survey so we can reach you. This information will be used for this purpose only, handled with discretion and erased afterwards.<br><br>If you have any questions regarding this research project, please feel free to contact *[AUTHORS]* (*[author@email.com]*) for further information.<br><br>Many thanks for your participation and support for this research project!<br><br>*[AUTHOR NAMES]* "
2	**[Attention test]**<br><br>"In this survey, you will view information about employees of a firm who work as software *engineers*. Your job is to read the information and estimate how successful an employee may be as a software *consultant* in the future in comparison to his/her colleagues.<br><br>Please read the instructions carefully. The closer your evaluations get to the employees' actual success as software consultants, the greater is your chance of winning an Amazon gift voucher. To test this, please only type the word experiment in the field below and ignore the following request to type the whole underlined sentence in the field.<br><br><u>It is important that you read the instructions thoroughly and carefully to obtain your chance of winning a voucher.</u>

	Please type the underlined sentence in the text box.” _____________________ (open input)
2a	**[If attention test = failed]**<br><br>“Unfortunately, it seems that you have not read the instructions carefully enough. For this experiment, it is very important to read the instructions thoroughly. In fact, it raises your chance to win the voucher.<br>Please try again!”<br><br>**[repeat screen No. 2]**
2b	**[If attention test = passed]**<br><br>“Well done, you passed the attention test! Thank you!<br>You may now continue and begin the experiment.”
3a	**[Experimental treatment]: Promotion [P_{pt}; P_{nt}]**<br><br>“Please imagine that you are the CIO (Chief Information Officer) of a big company that employs hundreds of software engineers. The business year has come to its end, and it is your choice who of your potentially best or, in other terms, who of your most promising software *engineers* you want to promote to software *consultants*.<br><br>As last business year’s sales were very pleasant, these promising employees’ training expenses will be raised. So, additionally to their salary increase because of the promotion, these employees will enter a very useful and prestigious mentoring program. The few mentees of this program are likely to occupy highly remunerated leading positions in the future. As part of the program, all mentees receive valuable amenities. For example, they are allowed to fly business class to any subsidiary worldwide at the firm’s expense – regardless of whether it is a business or private related flight.<br><br>Therefore, it is very important that you only choose your probably very best employees for this promotion round. To do this, you must evaluate every software engineer systematically and estimate how successful as a software consultant he or she could be.”
	**[Experimental task]: Promotion [P_{pt}; P_{nt}]**<br><br>“It is your specific job to estimate how successful your software *engineers* could be as software *consultants*.<br><br>Every business year, your company assesses the performance of a software consultant based on four equally weighted dimensions:<br>• The direct superior’s evaluation.<br>• The customer satisfaction (assessed on team level via a poll).<br>• The generated turnover from new and existing customers.<br>• The average delay in project due dates.<br><br>Your performance estimations will be matched with a score within a range from 1 to 100. For example, a score of 76 means that if *all* software engineers were promoted to software consultants, this employee would perform better than 75% of the rest. Accordingly, the best employees get 100 points and the worst 1 point.<br><br>**You will make 10 predictions. All employees above a certain threshold will be promoted.**<br>(All of the data shown in this study represents real data from real software engineers from different companies who, at a later date, at least once worked as software consultants.)”
3b	**[Experimental treatment]: Dismissal [D_{pt}; D_{nt}]**

<table>
<tr><td></td><td>
“Please imagine that you are the CIO (Chief Information Officer) of a big company that employs hundreds of software engineers. The business year has come to its end, and it is your choice whom of your potentially worst or, in other terms, least promising software engineers you want to dismiss.
Last business year’s sales declined severely and, though prior actions were undertaken, your company’s software engineer division is not competitive anymore. It must be closed, and dismissals are inevitable. Unfortunately, there are not enough means left to pay dismissal wages. Yet, some of your software engineers will be retrained as software consultants and can therefore stay, depending on their aptitude. All remaining software engineers will be dismissed.
Therefore, it is very important that you only choose your probably very worst employees for this dismissal round. To do this, you have to evaluate every software engineer systematically and estimate how successful as a software consultant he or she could be.”
</td></tr>
<tr><td></td><td>
[Experimental task]: Dismissal [D_{pt}; D_{nt}]
“It is your specific job to estimate how successful your software engineers could be as software consultants.
Every business year, your company assesses the performance of a software consultant based on four equally weighted dimensions:

The direct superior’s evaluation.
The customer satisfaction (assessed on team level via a poll).
The generated turnover from new and existing customers.
The average delay in project due dates.

Your performance estimations will be matched with a score within a range from 1 to 100. For example, a score of 76 means that if all software engineers were promoted to software consultants, this employee would perform better than 75% of the rest. Accordingly, the best employees get 100 points and the worst 1 point.
You will make 10 predictions. All employees below a certain threshold will be dismissed.
(All of the data shown in this study represents real data from real software engineers from different companies who, at a later date, at least once worked as software consultants.)”
</td></tr>
<tr><td>4</td><td>
“To make your predictions, you will see the following 8 criteria per employee. The data stems from last business year and is data about software engineers that your company collects on a regular basis. The values in square brackets are the lowest respectively highest values of all software engineers from last year.
Absent day(s) per business year
[0 – 26]
Work experience in years
[1 – 5 | 6 – 14 | 15–24 | 25–34 | ≥35]
Worked more than 2 years abroad
Number of ‘friends’ on the firm’s social intranet
[0 – 101]
Percentage of friends and relatives who also work in IT-related jobs
[0 – 10 | 11 – 20 | 21 – 30 | 31 – 40 | ≥ 41]
Ability to optimize code/runtime in relation to his/her colleagues
[Much worse | Worse | A little worse | Average | A little better | Better | Much better]
Has obtained a university degree?
</td></tr>
</table>

		**Average number of errors in script per 100 lines of code**<br>[0 – 20]<br><br>Accordingly, you will estimate for 10 software *engineers* how successful each of them could be as a software *consultant* next year, in each case using the 8 criteria above.<br><br>As a reminder, the performance score per software consultant is equally assessed in your firm based on 4 dimensions:<br>• The direct superior's evaluation.<br>• The customer satisfaction (assessed on team level via a poll).<br>• The generated turnover from new and existing customers.<br>• The average delay in project due dates."
5		**[Introduction to algorithm; P_{pt} and D_{pt} only]**<br><br>"In addition to the forecasts that you produce, there are also forecasts that are automatically produced by a statistical algorithm.<br><br>The algorithm is based on data from thousands of employees. It uses the same information for its predictions that you will receive and does not have any additional information that you will not receive. This is a sophisticated algorithm, put together by diligent analysts.<br><br>Before you make your 10 official predictions, you will go through 10 trial data sets to get used to the data.<br><br>You will also see the statistical model's ratings for each employee as well as the employee's true performance score as a software consultant of the following year.<br><br>*Please note: Due to technical reasons, the algorithm's predictions as well as employees' true performance score are displayed on the same screen (immediately after you typed your prediction in the box). Please mind that the system records if you alter your prediction 1 or more seconds after your initial input.*"
	5a-5j	**[Algorithm testing phase using 10 employee profiles randomly drawn from the 20 employee dossier sample; P_{pt} and D_{pt} only; <u>example (1 out of 20)</u>]**<br><br>"Absent day(s) per business year [0 – 26] — 2<br><br>Work experience in years [1 – 5 | 6 – 14 | 15 – 24 | 25 – 34 | ≥35] — 15 – 24<br><br>Worked more than 2 years abroad — No<br><br>Number of 'friends' on the firm's social intranet [0 – 101] — 57<br><br>Percentage of friends and relatives who also work in IT-related jobs [0 – 10 | 11 – 20 | 21 – 30 | 31 – 40 | ≥ 41] — 11 – 20<br><br>Ability to shrink necessary code/runtime in relation to his/her colleagues [Much worse | Worse | A little worse | Average | A little better | Better | Much better] — Average<br><br>Has obtained a university degree — Yes<br><br>Average number of errors in script per 100 lines of code [0 – 20] — 10<br><br>How successful do you think this employee will be as a software consultant?<br>(Please enter a score between 1 and 100.)<br><br>*For further information please scroll to the end of this site after your data input.*

		Algorithm's prediction: 47 **[number appears after participants' data input]**<br>Employee's actual performance score as a software consultant: 61" **[number appears after participants' data input]**
6	**[Introduction screen of main task]**	
	6a	**[P_$_{pt}$ only:]**<br>"Well done! The trial phase is over now.<br>After the following screen, your 10 official rounds start, in which you decide which of your employees you want to promote by estimating their performance scores.<br>Please do your best in submitting your 10 official estimates: The closer your submitted predictions are to the actual values the higher your likelihood of winning one of the three (1 x 75€, 1 x 50€, 1 x 25€) gift vouchers."
	6b	**[P_$_{nt}$ only:]**<br>"After the following screen, your 10 official rounds start, in which you decide which of your employees you want to promote by estimating their performance scores.<br>Please do your best in submitting your 10 official estimates: The closer your submitted predictions are to the actual values the higher your likelihood of winning one of the three (1 x 75€, 1 x 50€, 1 x 25€) gift vouchers."
	6c	**[D_$_{pt}$ only:]**<br>"Well done! The trial phase is over now.<br>After the following screen, your 10 official rounds start, in which you decide which of your employees you want to dismiss by estimating their performance scores.<br>Please do your best in submitting your 10 official estimates: The closer your submitted predictions are to the actual values the higher your likelihood of winning one of the three (1 x 75€, 1 x 50€, 1 x 25€) gift vouchers."
	6d	**[D_$_{nt}$ only:]**<br>"After the following screen, your 10 official rounds start, in which you decide which of your employees you want to dismiss by estimating their performance scores.<br>Please do your best in submitting your 10 official estimates: The closer your submitted predictions are to the actual values the higher your likelihood of winning one of the three (1 x 75€, 1 x 50€, 1 x 25€) gift vouchers."
7	**[Main task: The order of the two choice options is randomized between subjects]**<br>"Before you begin on the next screen, you can now choose for all of your 10 official predictions whether you want to save your own estimates or the statistical algorithm's estimates. In any case, both you and the algorithm will predict scores. This screen only determines which of the two ways of predicting you want to use for your 10 official submitted predictions.<br>Would you like to use your own estimates or the statistical algorithm's estimates to submit your 10 official predictions?<br>**[Option 1:]** ○ Use only the statistical algorithm's estimates for my 10 official predictions about the employee's future performance scores.	

	**[Option 2:]** ○ Use only my estimates for my 10 official predictions about the employee's future performance scores.<br><br>*Please note: You will also see the 10 estimates of the algorithm. Due to technical reasons these estimates are displayed on the same screen (immediately after you typed your prediction in the box). Please mind that the system records if you alter your prediction 1 or more seconds after your initial input.*"	
	7a-7j	**[Ten rounds of employee evaluation, drawn randomly from the remaining set of 10 employees from the 20-employees-sample; example:]**<br><br>Round [1 – 10] of 10.<br><br>Absent day(s) per business year [0 – 26] — 10<br><br>Work experience in years [1 – 5 | 6 – 14 | 15–24 | 25–34 | ≥35] — 15 – 24<br><br>Worked more than 2 years abroad — No<br><br>Number of 'friends' on the firm's social intranet [0 – 101] — 55<br><br>Percentage of friends and relatives who also work in IT-related jobs [0 – 10 | 11 – 20 | 21 – 30 | 31 – 40 | ≥ 41] — 21 – 30<br><br>Ability to optimize code/runtime in relation to his/her colleagues [Much worse | Worse | A little worse | Average | A little better | Better | Much better] — Average<br><br>Has obtained a university degree — No<br><br>Average number of errors in script per 100 lines of code [0 – 20] — 8<br><br>How successful do you think this employee will be as a software consultant? *Please enter a number between 0-100."* ______________<br><br>*For further information please scroll to the end of this site after your data input.*<br><br>Algorithm's prediction: 47" **[number appears after participants' data input]**<br><br>**[after 10 rounds of evaluation:]**<br><br>Well done, you passed the 10 rounds successfully! You may now continue.
8	**[Perceived confidence in algorithm and human forecast; 5-point Likert-type scale 1 = 'no confidence' to 5 = full confidence'; question order randomized between subjects:]**<br><br>1. "How much confidence do you have in the statistical algorithm's estimates?"<br>2. "How much confidence do you have in your own estimates?"	
9	**[McKnight et al. (2011): Trusting Stance & Faith in technology; 5-point Likert-type scale ranging from 1 = 'strongly disagree' to 5 = 'strongly agree']**<br><br>"Thank you very much!	

	You have completed the main part of the experiment. In the final part of this questionnaire, we would like to ask you seven questions on your personal preferences. Please reply spontaneously and be aware that there are no correct or incorrect answers.<br><br>Please indicate how much you agree or disagree with the following sentences:<br><br>1. I believe that most technologies are effective at what they are designed to do.<br>2. My typical approach is to trust new technologies until they prove to me that I should not trust them.<br>3. A large majority of technologies are excellent.<br>4. I generally do not give a technology the benefit of doubt when I first use it.<br>5. I think most technologies enable me to do what I need to do.<br>6. I usually trust a technology until it gives me a reason not to trust it.<br>7. Most technologies miss the features needed for their domain.”
10	**[Socio-demographics; dropdown options:]**<br><br>“Thank you!<br><br>In the last step, please provide us with the following demographics. Please indicate your…<br><br>- …year of birth? ___________________<br>- … gender? ___________________<br>- … highest level of education? ___________________<br>- … country of residence? ___________________
11	**[End of study and debriefing:]**<br><br>“This is the end of this study. Thank you very much for your participation! **Please do not forget to press the „Submit“-button at the end of this site.**<br><br>Please read the following debriefing of the experiment:<br><br>Actually, all employee data in this experiment is fictional and no algorithm was used to predict the performance of the employees. The main research question of this experiment addresses the circumstances under which people choose to let an algorithm make employee-related decisions instead of making them oneself.<br><br>As a result, there are no top three predicting participants that would be rewarded with gift vouchers. Instead, every participant may enter his/her email address and partake in a lottery to win one of the three vouchers, with equal chances for all who partake. Your email addresses will be handled with discretion, used only to contact you in case of winning and erased after all prizes have been paid out.<br><br>If you forward this experiment to someone else, please remain silent about its procedure and especially about the information on this page. Telling someone that it is “an experiment about employee evaluation”, for example, would be fine.<br><br>Best regards,<br><br>*[AUTHOR NAMES]*

Appendix B – Treatment and Ranking Mechanism

B.1 Construction criteria for generating a virtual sample of 25.000 software engineers in accordance with actual demographic data provided by Statistisches Bundesamt (2018)[1] *to maximize treatment credibility.*

Criterion	Values	Distribution M±SD	Manipulation	Weight
Day(s) absent per business year	0–26	normal 10.8±4	57x < 0 to 0	1.75 / 14
Work experience in years	0 = 1–5 y; 1 = 6–14 y; 2 = 15–24 y; 3 = 25–34 y; 4 = ≥ 35 y	normal 1.6±0.8	102x < 0 to 0<br>3x > 4 to 4	1.25 / 14
Worked more than two years abroad?	0 = No<br>1 = Yes	binomial 0.3	-	2.25 / 14
Number of 'friends' on the firm's social intranet	0–101	normal 50±18	56x < 0 to 0<br>47x > 101 to 101	2.5 / 14
Percentage of friends and relatives who also work in IT-related jobs	0 = [0–10%]; 1 = [11–20%]; 2 = [21–30%]; 3 = [31–40%]; 4 = [≥ 41%]	normal 1.5±0.8	174x < 0 to 0<br>2x > 4 to 4	1.75 / 14
Ability to optimize code / runtime in relation to colleagues [Likert-type scale]	0 = Much worse<br>6 = Much better	normal 3±1	9x < 0 to 0<br>6x > 6 to 6	1.25 / 14
Has obtained a university degree?	0 = No<br>1 = Yes	binomial 0.7	-	1.5 / 14
Average number of errors in script per 100 lines of code	0–20	normal 9±3	15x < 0 to 0	1.5 / 14
Random influence		unified	-1–1	0.25 / 14

[1] Statistisches Bundesamt. (2018). *Zahlen & Fakten – Indikatoren – Statistisches Bundesamt (Destatis): Qualität der Arbeit. Dimension 2: Einkommen und indirekte Arbeitgeberleistungen*. Retrieved from https://www.destatis.de/DE/ZahlenFakten/GesamtwirtschaftUmwelt/Arbeitsmarkt/_Doorpage/Indikatoren_QualitaetDerArbeit.html?cms_gtp=318944_slot%253D2

B.2 Ranking mechanism for sample of software engineers

**Criterion**	**How (high rank = better)**	**Values**	**Weight**	**Reason for weight with respect to being a software consultant**
Absent day(s) per …	1 – individual rank / max rank	0–1	1.75 / 14	It is relatively important to be 'constantly' available.
Work experience in …	individual rank / max rank	0–1	1.25 / 14	Important, but related to networking (see below).
Worked more than …	only dichotomous	0, 1	2.25 / 14	Intercultural experience increases necessary social skills.
Number of 'friends' …	individual rank / max rank	0–1	2.5 / 14	Networking is very important, hence highest weight.
Percentage of friends …	individual rank / max rank	0–1	1.75 / 14	Might facilitate gaining new customers.
Ability to shrink …	individual rank / max rank	0–1	1.25 / 14	Specifically important for a software engineer, not for a consultant.
Has obtained a …	only dichotomous	0, 1	1.5 / 14	Formal education mildly important.
Average number of …	1 – individual rank / max rank	0–1	1.5 / 14	Rather important for a software engineer, not for a consultant.
Random influence	unified distribution	-1–1	0.25 / 14	Reflects circumstantial factors that no one can account for. As the ranks were tied relatively closely together, 0.25 / 14 was chosen.

Note: The weighting mechanisms result in a percentile rank score ranging between 0 to 14 (min. to max.).

Appendix C – Model and Human Absolute Average Prediction Error (AAE), M (SD)

**Model AAE**	**All**	**Condition**		**Vignette**		**Treatment**			
		No-test	Pretest	Promotion	Dismissal	$P_{\_nt}$	$D_{\_nt}$	$P_{\_pt}$	$D_{\_pt}$
Before choice	20.33 (2.05)	-	20.33 (2.05)	19.98 (2.05)	20.62 (2.03)	-	-	19.98 (2.05)	20.62 (2.03)
After choice	19.86 (2.11)	20.04 (2.14)	19.66 (2.07)	19.96 (2.05)	19.78 (2.16)	19.9 (2.07)	20.17 (2.20)	20.02 (2.05)	19.36 (2.05)
Total	20.02 (1.56)	20.04 (2.14)	20.00 (0)	19.96 (1.54)	20.09 (1.58)	19.9 (2.07)	20.17 (2.20)	20.00 (0)	20.00 (0)
**Human AAE**									
Before choice	20.54 (6.65)	-	20.54 (6.65)	19.73 (7.31)	21.21 (6.01)	-	-	19.73 (7.31)	21.21 (6.01)
After choice	20.79 (5.52)	20.83 (5.27)	20.74 (5.81)	20.91 (6.26)	20.68 (4.74)	21.38 (6.05)	20.29 (4.34)	20.32 (6.52)	21.09 (5.14)
Total	20.71 (5.01)	20.83 (5.27)	20.58 (4.70)	20.77 (5.95)	20.66 (3.97)	21.38 (6.05)	20.29 (4.34)	20.02 (5.77)	21.05 (3.52)
**Different?**									
Before choice	No; t(153.42) = –0.34, p = .73	-	No; t(153.42) = –0.34, p = .73	No; t(67.04) = 0.26, p = .80	No; t(85.75) = –0.79, p = .43	-	-	No; t(67.04) = 0.26, p = .80)	No; t(85.75) = –0.79, p = .43
After choice	**Yes**; t(352.39) = –2.60, $\boldsymbol{p < .01}$	No; t(192.70) = –1.69, p = .09	No; t(158.67) = –1.97, p = .05	No; t(158.85) = –1.65, p = .10	**Yes**; t(198.53) = –2.07, $\boldsymbol{p < .05}$	No; t(88.66) = –1.97, p = .05	No; t(108.25) = –0.22, p = .82	No; t(69.31) = –0.34, p = .74	**Yes**; t(89.08) = –2.61, $\boldsymbol{p < .05}$
Total	**Yes**; t(326.68) = –2.19, $\boldsymbol{p < .05}$	No; t(192.70) = –1.69, p = .09	No; t(127.00) = –1.39, p = .17	No; t(148.45) = –1.54, p = .12	No; t(185.99) = –1.60, p = .11	No; t(88.66) = –1.97, p = .05	No; t(108.25) = –0.22, p = .82	No; t(58.00) = –0.03, p = .97	**Yes**; t(68.00) = –2.47, $\boldsymbol{p < .05}$

Dietvorst, B. J., Simmons, J. P., and Massey, C. (2015). Algorithm aversion: people erroneously avoid algorithms after seeing them err. *Journal of Experimental Psychology: General* 144, 114–126. doi: 10.1037/xge0000033.
